# What's in a tweet? Optimizing social media impressions

**DOI:** 10.1002/bco2.180

**Published:** 2022-08-04

**Authors:** Ralph Grauer, Dallin Busby, Emily Neckonoff, Mani Menon, Ketan Badani

**Affiliations:** ^1^ Department of Urology Icahn School of Medicine at Mount Sinai New York New York USA; ^2^ School of Medicine New York Institute of Technology College of Osteopathic Medicine Westbury New York USA

**Keywords:** engagement, social media, Twitter, visual abstract

The Internet has facilitated instant dissemination of information. In particular, the use of social media has fundamentally changed how scientific literature is engaged with and consumed. In 2017, most urologists were using social media, with 53% of survey responders reporting a Twitter profile.^1^ These accounts are used for various reasons, including professional networking, socializing, attending virtual journal clubs and debating topics on the academic frontier.^1^ Journals frequently share newly published works on social media, and professional societies—such as the European Association of Urology (EAU)—have used Twitter to boost awareness vis‐à‐vis guideline recommendations.^2^

Visual abstracts (VAs) have been created to present information in an easily digestible, visually appealing manner for social media posts. A VA is a pictorial representation of the background, methodology and key findings of a study. VAs typically accompany social media posts highlighting newly published articles. Preliminary work in the *British Journal of Urology International* (BJUI) reported a difference in likes and retweets between posts containing a VA and those without it.^3^ Herein, we build on that preliminary evidence by evaluating engagement (total likes, retweets and replies) and examining the factors associated with successful tweets, including the presence of VAs.

Our study aimed to determine the how the composition of a tweet affected engagement using the official BJUI Twitter account (@BJUIjournal). We analysed all tweets from November 2019 to October 2020, as this was the only era that contained VAs. In total, data on 421 tweets were extracted, 17 of which contained a VA. We compared engagement between tweets that contained a VA and those that did not. We found that the median engagement was 201 for those that contained a VA and 57 for that did not; the distributions in the two groups differed significantly (Mann–Whitney *U* = 777.0, *p* < 0.001). We then performed a linear regression to predict engagement using the following independent variables: presence of a VA, number of characters in the tweet, time of day (categorically defined as one of four 6‐h periods during a day) and number of hashtags. Because engagement (dependent variable) was not normally distributed, it was transformed into its logarithmic equivalent: log_10_(engagement). The assumptions of linear regressions held true: absence of multicollinearity, normally distributed residuals, homoscedasticity and no autocorrelation (Durbin–Watson test: 2.03). We found that VAs were positively associated with engagement (β = 0.601; *p* < 0.001) and the remaining variables were non‐significant (Table 1). The independent variable coefficients are interpreted as 1 unit increase results in a 10^β^ fold increase in engagement. Thus, the presence of a VA increases the engagement fourfold (10^0.601^). Overall, the model had an *R* ^2^ of 0.09 and was significant (*p* < 0.001).

**TABLE 1:** Linear regression predicting the log_10_(engagement)

	Beta	Standard error	*P* value
(Constant)	1.717	0.162	0.000
Visual abstract	0.601	0.100	0.000
Characters	0.000	0.000	0.757
Hashtags	−0.048	0.025	0.053
00:00–05:59 EST	0.114	0.223	0.608
06:00–11:59 EST	0.136	0.158	0.389
12:00–17:59 EST	0.075	0.153	0.627

Our study is unique as it investigates the composition and timing of a tweet to help optimize engagement. We find that VAs are the only significant factor associated with engagement. Though a modest 9% of the variation in engagement is explained by our model, we posit that this is substantial. Our model explains nearly 10% of a tweet's engagement without consideration of the scientific content, especially considering there is only one statistically significant variable. The benefit we found in leveraging VAs aligns with previously reported data involving randomized trials and crossover trials investigating the role of VAs.^4^, ^5^, ^6^ In the most robust analysis of VAs in urology published to date, Klaassen et al. performed a prospective randomized trial comparing the accompaniment of either VAs or key figures to tweets promoting manuscripts.^4^ They report that VAs increased Twitter impressions compared with tweets with only images of key findings, but VAs decreased the amount of traffic to the full article via an embedded link. In other words, VAs increased the reach of the literature, but decreased the depth of these interactions. Thus, the authors recommend that VAs be used judiciously with consideration for the value of a full‐article view compared with engagement with the abstract. Linking increased Twitter exposure to the tangible endpoint of increased article citations, Sathianathen et al. found that social media and other online factors can help predict 2‐year citations of an article with an *R* ^2^ of 0.14.^7^ There is little doubt of the benefit of using VAs to increase exposure.

Though our findings align with previously reported data on the positive influence of VA, there are notable limitations. First, the analysed tweets were not randomized for VA inclusion; thus, it is the possible the VAs were produced for studies that are inherently more interesting. This confounds the interpretation of the tweets' engagement. We also did not exclude non‐VA tweets that did not promote a manuscript, a decision that may artificially deflate engagement. Importantly, our sample size was relatively small, composed of 421 tweets, and only 17 (4%) contained a VA. Though largely confirmatory, these findings uniquely examine the composition of a tweet in addition to merely the presence of a VA. Unlike Shakespeare's famous quote in *Romeo and Juliet*: ‘What's in a name? That which we call a rose by any other name would smell as sweet’, we conclude that tweets are indeed much sweeter if they include a VA.

## CONFLICT OF INTEREST

None of the authors have any relevant disclosures, and none of the authors have any financial or non‐financial interests that may be relevant to the submitted work.
